# Jaw osteosarcoma and pregnancy: a rare coexistence

**DOI:** 10.4322/acr.2021.359

**Published:** 2022-02-24

**Authors:** Thaís Aguiar Santos, Márcia Gimenes Américo, Antonio Vitor Martins Priante, Maria Fernanda de Oliveira, Ana Lia Anbinder

**Affiliations:** 1 Universidade Estadual Paulista (UNESP), Institute of Science and Technology, Department of Biosciences and Oral Diagnosis, São José dos Campos, SP, Brasil; 2 Prefeitura de Taubaté, Oral Health Division, Taubaté, SP, Brasil; 3 Hospital Regional do Vale do Paraiba, Taubaté, SP, Brasil; 4 Universidade de Taubaté, Department of Medicine, Taubaté, SP, Brasil

**Keywords:** Osteosarcoma, Pregnancy, Bone Neoplasms

## Abstract

Osteosarcoma of the jaw represents less than 1% of all head and neck malignancies. This malignancy in pregnant women occurs in one per 1000 deliveries. We report a case of a 29-year-old woman, in the 33rd week of gestation, who presented with an expansive tumor destroying the maxillary alveolar bone, histologically composed of pleomorphic, round, spindle, or epithelioid cells and osteoid/chondroid matrix. Upon final diagnosis of osteosarcoma, the lesion was excised. To the best of our knowledge, only 10 cases of jaw osteosarcoma in pregnant women have been reported to date in the English language literature. The use of ancillary examinations, malignancy diagnosis, and cancer treatment can be challenging during pregnancy. Knowledge about jaw osteosarcoma in pregnancy can increase healthcare providers’ awareness, avoid delays and misdiagnosis and potentially improve maternal and neonatal outcomes.

## INTRODUCTION

Osteosarcoma is a malignant and aggressive bone tumor characterized by osteoid tissue or immature bone by tumor cells.^1^ It frequently affects long bones in the appendicular skeleton, such as the femur. It is more prevalent in children and teenagers than in adults, with a higher incidence in the male population.^2^

Osteosarcoma in the head and neck region is singular and unusual, accounting for approximately 1% of the malignancies that affect these regions.^3^ The incidence rate of primary craniofacial osteosarcoma is 0.39 per million.^4^ It occurs mainly in the jaws, affecting the mandible slightly more than the maxilla. Its characteristics differ from osteosarcoma of the long bones in several aspects, such as age, grade of malignancy, and prognosis. Osteosarcoma occurs in an older age group in the jaw and often shows little cellular atypia, less aggressiveness, and late metastasis. Consequently, several authors considered it a distinct entity, different from osteosarcoma of the long bones.^4^

Malignancy in pregnant women is also uncommon and occurs in one per 1,000 deliveries.^5^ Recently, pregnancy-associated cancers have increased, partially explained by the tendency to delay pregnancy until later, and possibly associated with improved diagnostic techniques, detection, and increased health services access.^5^ Breast, skin, lymphohematopoietic, and gynecological cancers are among the most frequent pregnancy-associated malignancies,^5^^,^ 6 whereas head and neck cancer account for 0.9% of them.^6^

Cancer diagnosis in a pregnant patient may be challenging since there are overlapping symptoms common in pregnancy and malignant disease; there are also insecurities in the safety of several ancillary examinations in this population. Delay in diagnosis and treatment occurs in 65% of pregnancy-related cancer cases and is mainly caused by healthcare providers.^7^ In a retrospective study, considering the occurrence of musculoskeletal tumors in women, only 3.33% of the cases were diagnosed during pregnancy. Each pregnant patient was misdiagnosed at least once, and the time for diagnosis was significantly higher for pregnant than non-pregnant patients.^8^

We present a rare case of conventional osteosarcoma in the maxilla of a pregnant woman and a literature review. The diagnostic difficulties in such cases were discussed to increase the clinicians’ awareness regarding this uncommon condition.

## CASE REPORT

**Figure 1:**
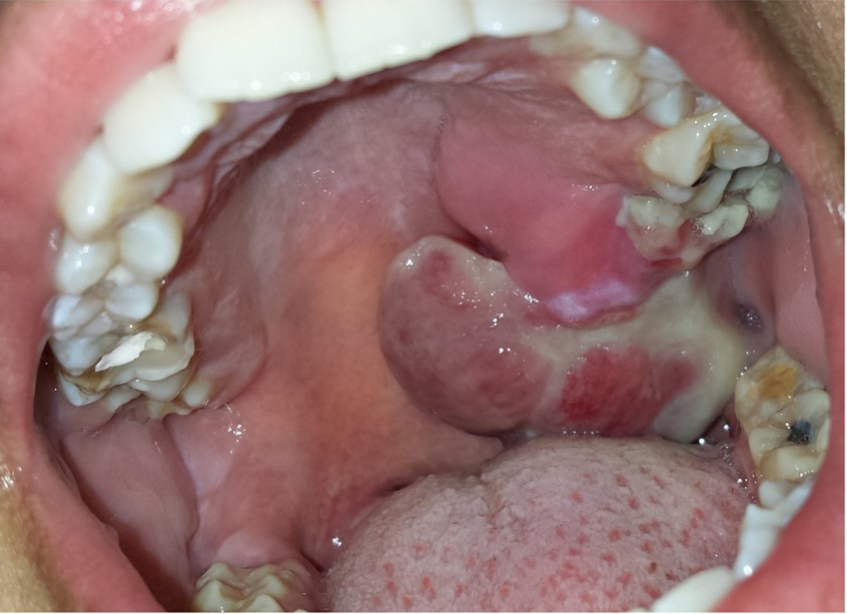
Gross view of the lesion at the first appointment. Extensive reddish, multilobulated, ulcerated and nodule in the left posterior maxilla, involving hard and soft palates, can be seen.

A 29-year-old black woman, in the 33rd gestation week, presented with slight facial asymmetry and an extensive reddish, multilobulated, ulcerated, and painful nodule in the left posterior maxilla, involving the hard and soft palates (Figure 1), that began 1 month ago. In addition to pain, paresthesia of the dorsum of the tongue was reported by the patient.

**Figure 2:**
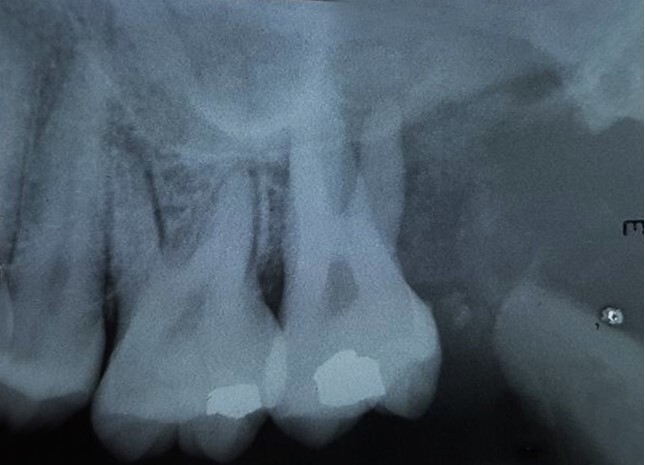
Periapical radiograph, showing bone destruction in the area of the absent third molar and around the second molar, reaching the maxillary sinus, and widening of the space occupied by the periodontal ligament around the first molar.

The submandibular and cervical lymph nodes were palpable and painful, and the first and second molar teeth enwrapped in the lesion presented great mobility. Bone destruction was found in the area of the missing third molar and around the second molar, reaching the maxillary sinus. Moreover, widening of the space occupied by the periodontal ligament around the first molar could be seen on the periapical radiograph (Figure 2).

Computerized tomography (CT) also showed an expansive hypodense tumor destroying the alveolar bone around the molars, which invaded the maxillary sinus (Figures 333C), with apparent extension to the left oropharynx.

**Figure 3:**
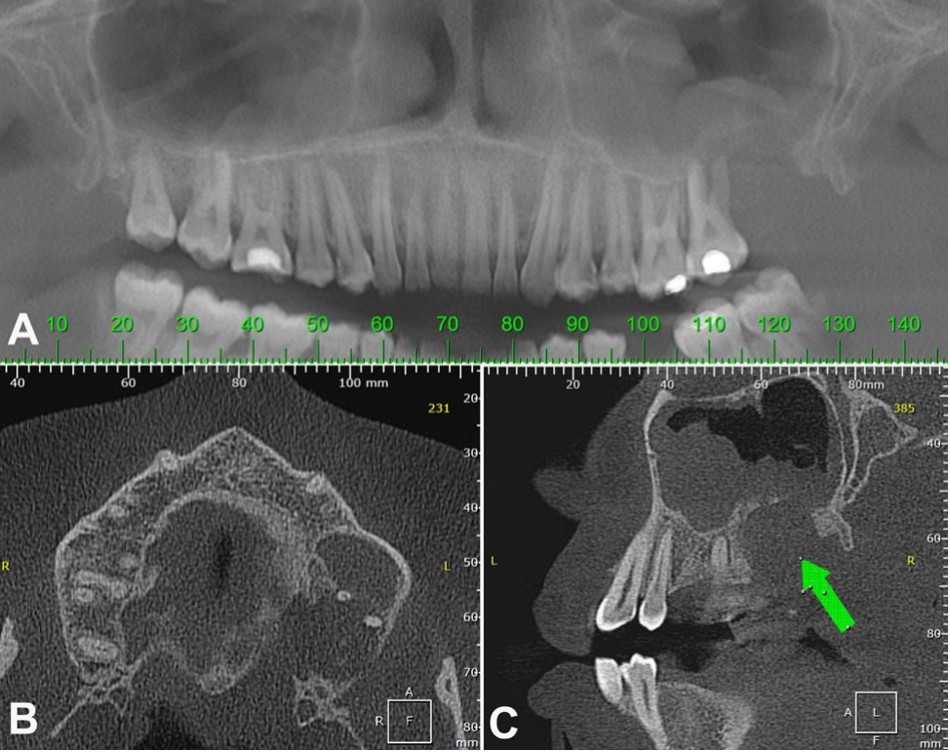
**A** – Panoramic reconstruction from computerized tomography, where the extension of the lesion can be evaluated better. Widening of the space occupied by the periodontal ligament around the first molar is also noted; **B** – Computerized tomography axial plane, showing an expansive hypodense tumor destroying the alveolar bone and expanding the maxillary cortical bone; **C** – Computerized tomography sagittal plane, showing an expansive hypodense tumor (arrow) invading the maxillary sinus.

**Figure 4:**
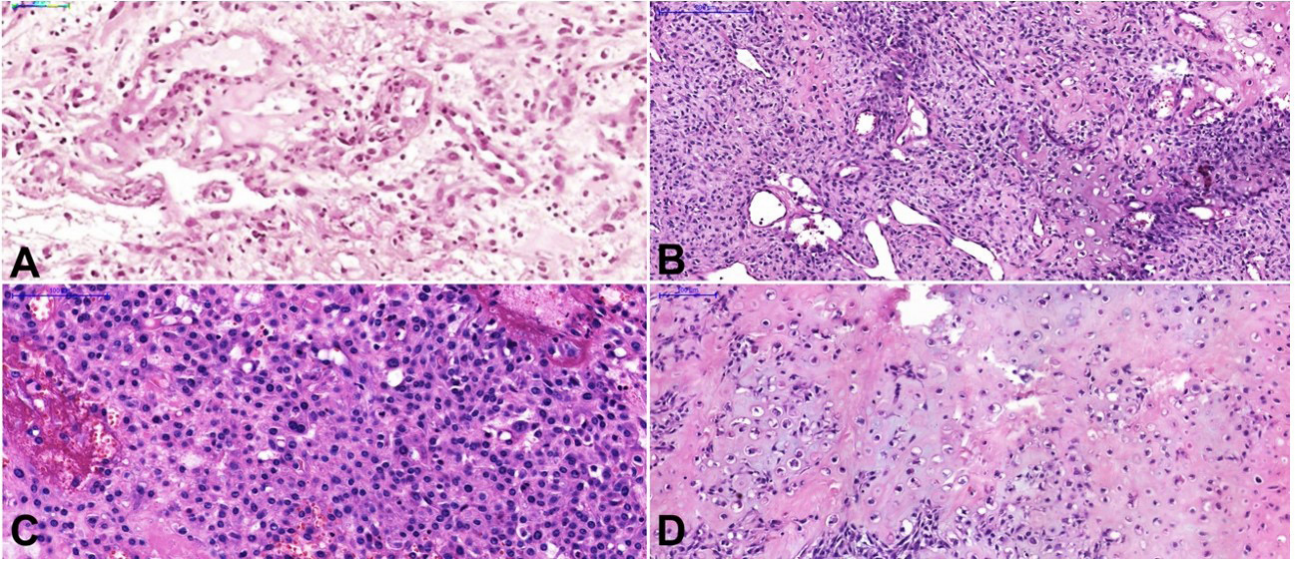
Photomicrographs of the lesion. **A** – Epithelioid cells, with eosinophilic cytoplasm and a small amount of eosinophilic matrix, in a very well-vascularized stroma could be seen in the first biopsy (H&E, Scale bar=50µm); **B** – The tumor, extremely vascularized, was composed of pleomorphic cells among the osteoid (upper left) or chondroid (bottom right) matrix (H&E, Scale bar=200µm); **C** – Round epithelioid cells with prominent cytoplasm and pleomorphic nuclei, sometimes multinucleated (H&E, scale bar=100µm); **D** – Chondroid and osteoid matrix are sometimes intermingled (H&E, scale bar=100µm). All the slides were digitized using a whole slide scanner (Pannoramic Desk, 3DHistech), with x20 objective.

An incisional biopsy was performed with a clinical hypothesis of salivary gland neoplasm and giant cell granuloma. The small fragments were covered by a thick fibrin exudate, and the connective tissue presented abundant edema and intense inflammation. Epithelioid cells, with eosinophilic cytoplasm, pleomorphic nuclei with clear chromatin, and central nucleoli were found, in addition to spindle cells and a small amount of eosinophilic matrix, in a very well-vascularized stroma (Figure 4A).

Immunohistochemical reactions were performed for AE1/AE (AE1/AE3, dilution 1:50, Dako), epithelial membrane antigen (E29, dilution 1:50, Dako), HMB45 (HMB45, dilution 1:50, Dako), and vimentin (V9, dilution 1:400, Dako) antibodies, and the lesion cells were positive only for vimentin. Although it was suggestive of osteosarcoma, the biopsy was superficial, with an extensive ulcerated area covered by necrosis, in addition to an intense inflammatory infiltrate. Despite the clinical and imaging features compatible with osteosarcoma, the biopsy material was considered insufficient to conclude the histopathological diagnosis; thus, another biopsy was recommended.

The disease progression was rapid and resulted in feeding difficulty. Before the second biopsy, at 36 weeks of pregnancy, labor was induced, and the patient gave birth to her fifth child, a healthy boy weighing 6.63 pounds through vaginal delivery.

**Figure 5:**
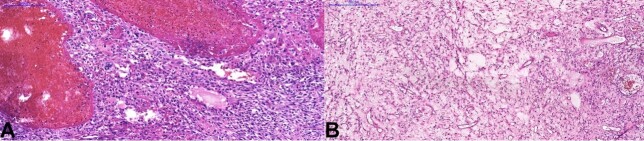
Photomicrographs of the lesion. **A** – Telangiectatic areas and pleomorphic spindle cells (H&E, scale bar=200µm); **B** – Highly vascularized myxoid areas. H&E, scale bar=500µm). All the slides were digitized using a whole slide scanner (Pannoramic Desk, 3DHistech), with x20 objective.

A second biopsy was then performed at another medical center, and the lesion was misdiagnosed as a pyogenic granuloma. The patient was then subjected to conservative excision of the lesion. Histologically, the tumor was composed of round cells with prominent cytoplasm and pleomorphic nuclei, sometimes multinucleated. Areas with spindle and epithelioid cells were also found admixed with osteoid or chondroid matrix. The lesion was extremely vascularized, with myxomatous and telangiectatic regions (Figure 4 and 5). The final diagnosis was conventional osteosarcoma, and the patient underwent another procedure to expand the surgical margins. The margins and local lymph nodes were free of neoplasia. A microsurgical flap from the left forearm was used for reconstruction. The patient received adjuvant radiation (6000 cGy in 30 sessions) and chemotherapy (6 cycles of cisplatin (100 mg/m^2^), adriamycin (75 mg/m^2^), and growth factor support with filgrastim). She was followed up for 4 years, with no signs of metastasis or recurrence. Informed consent was obtained from the patient.

## DISCUSSION

Diagnosing malignancy during pregnancy is rare and challenging owing to the several overlapping symptoms of pregnancy and malignancy, the uncertainties of the health care team about the safety of several diagnostic tests, and the difficulties in histopathologic diagnoses.^9^

Primary osteosarcoma in the jaws during pregnancy is also a rare event. A few cases have been described in the literature to date. An electronic search was performed in June 2020 in the PubMed/Medline, Scopus, Web of Science, and Google Scholar databases, using the keywords “osteosarcoma” and “jaw” and “pregnancy,” with no date restriction. The references cited in several papers were also searched. Ten published cases were reported in English (Table 1).^10^^-^ 18

**Table 1:** Review of reported cases of jaw primary osteosarcoma in pregnancy in the English-language literature

Ref	Age	Site	Trimester of pregnancy when osteosarcoma was diagnosed		Histological type	Treatment	Delivery and condition of the babies
10	NA	Mandible	3rd	NA		Surgery	normal
10	NA	Mandible	2nd	NA		Surgery, radiation therapy	Full term, normal
11	38	Mandible	3rd	Chondroblastic and osteoblastic		Surgery	Full term, cesarian birth, normal
12	28	Mandible	1st	Small cell		Surgery and Chemotherapy	Therapeutic abortion
13	38	Mandible	NA	Chondroblastic		NA	NA
14	22	Maxilla	3rd	Epithelioid		NA	NA
15	25	Mandible	2nd	Pleomorphic		Surgery, Radiation and Chemotherapy	Therapeutic abortion
16	31	Maxilla	3rd	Chondroblastic		Neoadjuvant chemotherapy and Surgery	Pregnancy interrupted in the 33^rd^ week
17	NA	Mandible	3rd	Chondroblastic		Surgery, radiation therapy	Induced delivery at 35 weeks
18	20	Mandible	1st	Chondroblastic		Surgery, NA	Pregnancy interrupted, NA
Index case	29	Maxilla	3rd	Chondroblastic		Surgery, Radiation and Chemotherapy	Induced delivery, full term, normal

Ref= reference.

The mean age of patients diagnosed with jaw osteosarcoma during pregnancy was 28.88 ± 6.69 years, 72.73% of cases occurred in the mandible, 60% were diagnosed during the 3^rd^ trimester of pregnancy, 66.67% of the known histological subtype was chondroblastic, and the main treatment was surgery (100% of cases, when the information was available).

The diagnostic approach for bone lesions requires a correlation between clinical, imaging, and histopathological examination (the triple diagnostic approach). X-rays, CT scans, or magnetic resonance imaging can offer important information, such as tumor margins, areas of bone destruction, involvement of the bone cortex, and pathological fractures^19^. In our case, the clinical diagnostic hypothesis was giant cell granuloma and salivary gland neoplasm. Based on the clinical features, on the enlargement of the periodontal ligament and bone destruction in the radiograph, the hypothesis of osteosarcoma could have been raised at that time. Widening of the periodontal ligament space of the involved teeth is considered the earliest radiographic manifestation of osteosarcoma involving the jaws.^20^ However, periosteal reaction, found in 62% of jaw osteosarcomas, was not present in this case.^21^ Moreover, despite the importance of the imaging examination, some clinicians are reluctant to refer a pregnant patient for a radiograph or CT examination, which could also cause a diagnostic delay. Ionizing imaging is safe during pregnancy when a fetal radiation threshold of 100mGy is maintained. Excluding a CT scan of the pelvis, all ionizing techniques remain below 100mGy and are considered safe.^9^

Neurologic deficit and paresthesia are not uncommon in cases of jaw osteosarcoma.^20^ In our case, the lesion could have affected the glossopharyngeal nerve, leading to tongue paresthesia. Several other clinical hypotheses were found in the literature before the final diagnosis of jaw osteosarcoma, such as a periapical abscess,^22^ cemento-osseous lesions,^23^ pyogenic granuloma^16^, osteomyelitis, and several malignancies, such as sarcomas (chondrosarcoma, fibrosarcoma, leiomyosarcoma, Ewing’s sarcoma) and bone metastasis.^24^ Pyogenic granuloma is a benign vascular tumor also known as a pregnancy tumor when seen in pregnant patients. It occurs in the gingiva of 2.15% of pregnant women and is caused by irritant agents, such as bacterial biofilms under increased hormonal levels.^25^ This lesion is well known by general clinicians, occurring in pregnant women with an average age of 28. In 52.22% of the cases, the lesion appeared in the third trimester of gestation.^26^ It can present a fast growth pattern, which may be worrisome to patients and clinicians, but generally does not affect the bone. Histologically, atypia due to ulceration and reactive changes may be pronounced in pyogenic granuloma,^9^ and this must be considered during diagnosis. The lesion in the present case was well-vascularized, but the previous histological and clinical characteristics were not considered in the diagnosis of the second biopsy, leading to a misdiagnosis. Caution should be exercised to avoid misdiagnosing a rare, well-vascularized malignant bone lesion as a common pyogenic granuloma, delaying diagnosis and treatment, as in the present case.^16^

The microscopic diagnosis of osteosarcoma is based on malignant tumor cells and neoplastic bone. However, it may be challenging, especially in small tissue biopsies and small amounts of osteoid, since osteosarcoma has several histologic variants, and its features can mimic other malignancies. In the presence of abundant osteoid, the diagnosis is straightforward; however, in fibroblastic areas, it may be confused with collagen, and when extensive chondroid differentiation is present, it can be misdiagnosed as chondrosarcoma.^27^ In such cases, the use of immunohistochemical markers may be helpful. Alkaline phosphatase immunohistochemistry is useful in discriminating osteosarcoma cells from other primary bone tumors,^28^ such as angiosarcoma and low-grade fibromyxoid sarcoma. Although not specific for osteosarcoma, SATB2, a marker of osteoblastic differentiation, is a useful adjunct in distinguishing between hyalinized collagen and osteoid. Antibodies against osteonectin and osteocalcin have already been evaluated in this scenario, with relatively low sensitivity and/or specificity.^29^ Unfortunately, none of these markers were available at the time of the first biopsy.

Multidisciplinary teamwork is needed to define the best patient-centered approach to treat cancer during pregnancy. Biopsies and surgical procedures are relatively safe when needed in pregnant women and should not be delayed if indicated.^30^ The best treatment strategy and pregnancy interruption must be decided after balancing the risks to the mother and fetus. The gestational period, the location, type and grade of the tumor, threat to the mother's life, with or without treatment, and risks to fetal safety as an impact on growth, maturation, development, and even death must be taken into account.^30^ In the present case, labor was induced at 36 weeks, even before the final diagnosis, due to feeding impairment caused by the lesion.

Surgical treatment resection with adequate margins is the main choice for osteosarcomas, with better results achieved with adjuvant radiotherapy and chemotherapy to improve local control of the disease and overall survival. Most cases of recurrence are found over a 5-year period.^3^ The patient in the present case underwent a local resection with reconstruction using a microsurgical flap and received adjuvant radiation and chemotherapy thereafter.

Pregnancy is a proangiogenic state, and hormones and growth factors important for fetal development may increase tumor development.^5^ Several cancers have been linked to increased estrogen levels, such as breast, ovarian, gastric, pituitary, and thyroid cancers,^31^ and several tumors have been identified as growing faster in pregnant woman than in other patients. 32 Estrogen receptor (ER)-β and progesterone receptor (PR) were present in the majority of osteosarcoma cases analyzed by Dohi et al.,^33^ but ER-α and aromatase were not detected. The same authors found that the proliferation of osteosarcoma cells was stimulated by estradiol and progesterone and suppressed by ER and PR blockers. Interestingly, Dominguez-Malagón et al.^34^ found negative expression of ER and PR in 95.24% and 100% of the cases of jaw-osteosarcoma, respectively. They attribute such differences to ossification mechanisms and bone metabolism in the jaws. The relationship between pregnancy and malignant changes remains unclear and requires further study.

## CONCLUSION

Although pregnancy can influence tumor growth, it increases women’s interaction with health services and the possibility of diagnosis.^5^ Raising awareness regarding cancer in pregnancy among healthcare providers may help avoid delays in the diagnosis and therapy, potentially improving maternal and neonatal outcomes.^7^
